# Near-Field Coupling with a Nanoimprinted Probe for Dark Exciton Nanoimaging in Monolayer WSe_2_

**DOI:** 10.1021/acs.nanolett.3c00621

**Published:** 2023-06-01

**Authors:** Junze Zhou, John C. Thomas, Elyse Barre, Edward S. Barnard, Archana Raja, Stefano Cabrini, Keiko Munechika, Adam Schwartzberg, Alexander Weber-Bargioni

**Affiliations:** † The Molecular Foundry, Lawrence Berkeley National Laboratory, 1 Cyclotron Road, Berkeley, California 94720, United States; ‡ HighRI Optics, Inc. 5401 Broadway Ter 304, Oakland, California 94618, United States

**Keywords:** tip-enhanced photoluminescence, gap-mode plasmonic cavity, dark exciton, 2D materials, nanobubble

## Abstract

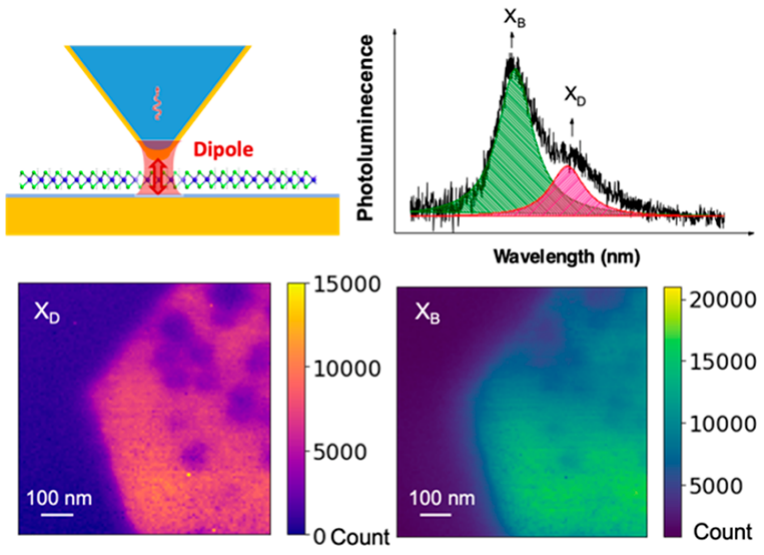

Tip-enhanced photoluminescence (TRPL) is a powerful technique for spatially and spectrally probing local optical properties of 2-dimensional (2D) materials that are modulated by the local heterogeneities, revealing inaccessible dark states due to bright state overlap in conventional far-field microscopy at room temperature. While scattering-type near-field probes have shown the potential to selectively enhance and reveal dark exciton emission, their technical complexity and sensitivity can pose challenges under certain experimental conditions. Here, we present a highly reproducible and easy-to-fabricate near-field probe based on nanoimprint lithography and fiber-optic excitation and collection. The novel near-field measurement configuration provides an ∼3 orders of magnitude out-of-plane Purcell enhancement, diffraction-limited excitation spot, and subdiffraction hyperspectral imaging resolution (below 50 nm) of dark exciton emission. The effectiveness of this high spatial X_D_ mapping technique was then demonstrated through reproducible hyperspectral mapping of oxidized sites and bubble areas.

Tip-enhanced photoluminescence (TEPL) nanospectroscopy and -imaging is a demanding technique that enables spectrally and spatially resolved emission of materials at length scales beyond the diffraction limit.^1^ Since the first demonstration by Betzig et al.^2^ of single-molecule detection using an aperture-type near-field probe, advanced near-field probes (configurations) with improved throughput have been proposed to study new luminescent nanomaterials. Among them, two types of near-field probes, including the “Campanile” probe^3^ with in-plane near-field enhancement and probe–substrate-based gap mode plasmonic^4^ with sensitivity in the out-of-plane direction have been successfully employed to optically map the subdiffraction limit localized excitonic states in two-dimensional (2D) transition-metal dichalcogenides (TMDs)^5,6^ across local heterogeneities such as point defects,^7,8^ twin boundaries,^4^ and localized strain.^9−11^

Recently discovered spin-forbidden dark excitons (X_D_)^12−14^ in 2D TMDs have long radiative lifetimes^15,16^ and are the excitonic ground state in tungsten-based TMDs, making them attractive for quantum information applications.^16−19^ The X_D_ exciton dipole is oriented out of plane and orthogonal to the bright exciton (X_B_),^20^ making it accessible at room temperature through out-of-plane near-field coupling.^12,13,16,17,21,22^ Gap-mode plasmonic cavity enhancement has been suggested to selectively isolate X_D_ emission at room temperature.^21,22^ However, to the best of our knowledge, reproducible and robust direct spectral and spatially resolved TEPL of dark excitonic properties over micrometer-scale dimensions has yet to be fully demonstrated. Consequently, the impacts of the nanoscale heterogeneities on the X_D_ have much information to be unveiled.

Here, we demonstrate a unique TEPL configuration using a nanoimprinted fiber probe with a thin plasmonic coating that, when coupled to a gold surface, enables X_D_ nanoimaging in monolayer (ML) WSe_2_. This gap-mode plasmonic cavity couples out-of-plane X_D_ emission into the near-field probe and attached optical fiber, allowing for direct observation at resolutions on the order of the probe radius (tens of nanometers).^21,22^ The in-plane bright state, on the other hand, is not enhanced due to polarization mismatch, while the out-of-plane Purcell cavity selectivity enhances the dark state emission by ∼3 orders of magnitude, enabling high spatial resolution hyperspectral mapping of local heterogeneities such as nonemissive oxidized sites and nanobubble areas. This efficient nanoimaging configuration provides direct insights into the impact of chemical modifications and localized strain on the dark states.

As shown in Figure 1a, the near-field probe is based on a sharp pyramid placed onto the end facet of a single-mode optical fiber (630HP, Thorlabs) using nanoimprint lithography.^23^ With tuning-fork feedback, the pyramid probe provides excellent topographic sensitivity and a confined light intensity profile.^23,24^ To enhance out-of-plane dark excitonic emission, a gap mode plasmonic cavity was fabricated by coating the probe with a ∼20 nm gold thin film and the substrate with a 100 nm gold film to support the ML WSe_2_. The sample was separated from the substrate gold by ∼2 nm of atomic layer deposition (ALD) grown SiO_2_ to reduce Ohmic contact and photoluminescence quenching (for more details, see Section S1 in the Supporting Information).^25^ The spontaneous decay rate of a quantum emitter can be significantly modified by a photonic cavity through the Purcell effect, where the Purcell factor *F*_P_ is inversely proportional to , where *V* is the effective mode volume. In the probe-based gap mode configuration the mode volume scales with the gap spacing.^26^ The enhanced emission signal in the lateral direction is determined by the radius of the probe apex, while the signal in the out-of-plane direction can be dynamically controlled by changing the probe–sample distance.^21^ The strong polarity of the gap mode results in several orders of magnitude stronger near-field enhancement for the out-of-plane dipole emission than for that of the in-plane emission.^22^

**Figure 1:**
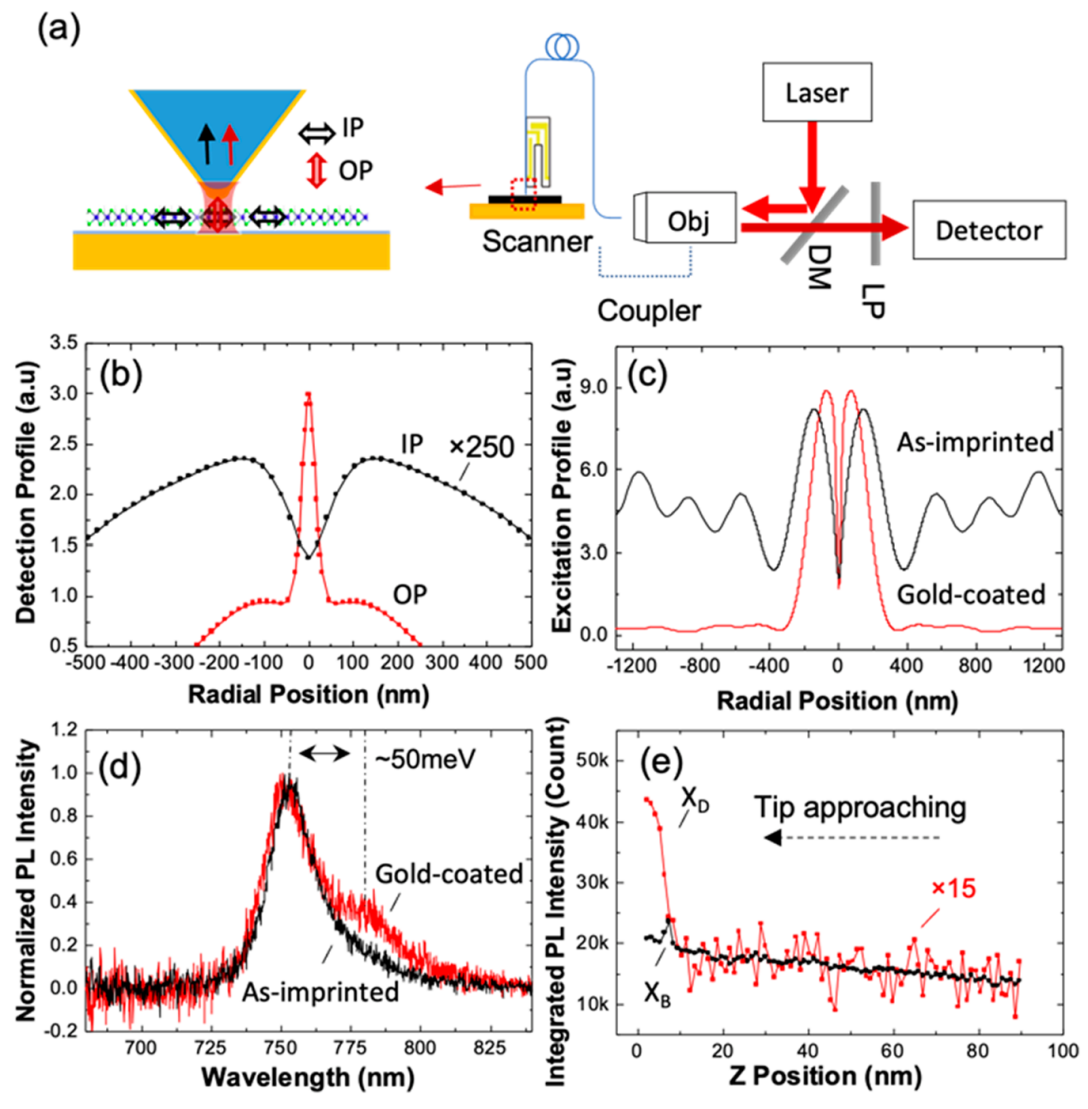
A fiber probe based scanning near-field microscope for X_D_ mapping through out-of-plane gap-mode plasmonic enhancement. (a). Left: the enhancement scheme of out-of-plane X_D_ through the gap mode configuration composed of the gold pyramid probe and the gold substrate. OP and IP denote out-of-plane and in-plane, respectively. Right: schematic of the optical measurement configuration. The excitation laser is coupled to the fiber probe, and the PL signal is collected back through the probe. (b) Numerical simulations of the collection profiles of the in-plane (IP) and out-of-plane (OP) dipoles swept across the gap on the sample plane. The simulated intensity of the IP dipole was multiplied by 250 times for comparison purposes. (c) Numerical simulations of the excitation laser intensity at the sample plane. (d) PL spectra of the ML WSe_2_ collected through the gold pyramid probe in comparison with that collected through the bare pyramid probe. The dark state is found at ∼50 meV below the bright exciton. (e) Probe–sample distance dependence of the integrated intensity of X_D_ and X_B_. The value of X_D_ was multiplied by 15 times for comparison purposes.

The measurements were conducted using a AIST-NT TRIOS scanning probe microscope, where the probe–sample distance was regulated by the tuning fork operating in shear-force mode.^27^ A low-power (<20 μW) 632.8 nm He–Ne laser was coupled into the fiber (10×, 0.25 NA), as shown in Figure 1a, and guided to the sample through the probe. The emission was coupled back through the same path, separated from the excitation source by a dichroic mirror (LP02-633-RE-25, Semrock), and sent to a spectrometer (Kymera 328i, Andor) for analysis. Hyperspectral PL maps were obtained by integrating the spectrum for 200 ms at each point.

The resolution of a TEPL system is determined by both the collection and excitation spot sizes, which were first simulated using Lumerical FDTD (see Section S2 in the Supporting Information for more details). For the detection profile, a single dipole was scanned across the sample surface, and the collected intensity was monitored in the probe. Figure 1b shows the cross-section of the detection profile of the out-of-plane and in-plane dipoles. The out-of-plane dipole was confined to a full width at half-maximum (fwhm) of ∼30 nm, while the in-plane dipole case was much larger, on the scale of hundreds of nanometers. The calculated excitation profiles presented in Figure 1c reveals that the gap-mode configuration provides a highly confined excitation spot, with a size of hundreds of nanometers, which is close to the diffraction limit at the excitation wavelength without the need for a high numerical aperture (NA) objective lens.^28^ This high NA property of the probe is primarily determined by the optical cavity and tapered angle of the nanoimprinted pyramidal probe.^23^ This confined excitation spot is critical for probing the dark state emission, as it minimizes the amount of bright excitons that are excited, which can act as background noise and obscure the signal from the dark states in the room-temperature spectra. In contrast, the scattering-type near-field technique typically has a far-field spot size coupled from the side objective lens typically on the order of micrometers,^10^ leading to a larger amount of bright excitons being excited. Moreover, the addition of a gold coating to the pyramid probe not only forms the gap mode with the gold substrate but also helps to reduce the relative intensity of the sidebands beyond the apex of the probe. This is attributed to the more efficient coupling of evanescent energy toward the apex of the probe through the gold coating, which improves the spatial resolution and sensitivity of the TEPL technique.

The strong polarization collection anisotropy is the key feature of this TEPL technique, which enables nanoscopic imaging of dark states. By utilizing the out-of-plane probe–sample coupling, the technique significantly enhances the out-of-plane polarized dark state emission, while the in-plane polarized bright state emission is not enhanced. The plasmonic coupling also limits the collection volume of the dark state emission, resulting in extremely high imaging resolution. In contrast to scattering-type near-field techniques,^10,29^ this novel near-field configuration offers a reduced interaction between the excitation laser and the probe, which minimizes potential instability factors such as the probe wear,^30^ electrostatic potential,^31^ and thermal drift.^28^ The heating effect generated by the excitation laser can be significantly reduced, by up to 5 orders of magnitude, through the combination of reduced near-field enhancement, as discussed above, 2 orders of magnitude larger spatial localization of excitation laser at the probing region. This promotes stable and consistent near-field measurements, while also addressing the challenges encountered during TEPL mapping of 2D materials, which have been reported to be sensitive to light^32^ and electrostatic potential^33^ under ambient conditions. The approach leverages the advantages of collection mode scanning near-field microscopy (SNOM),^34^ generally known for causing less probe wear compared to the illumination mode. Furthermore, it is operated in reflection mode by delivering light through the probe, allowing for great flexibility and ease of use.

The PL emission of the WSe_2_ sample was initially measured using the pyramidal probe with and without gold coating to evaluate the effect of the gap mode. The results in Figure 1d indicate that with the uncoated probe, only a single bright exciton feature is detected, whereas a new feature at approximately 50 meV red-shifted from the bright exciton band appears upon adding the gold coating. This energy difference is in agreement with the previous reports of dark state emission.^19,21,29^ The integrated PL intensity of these two peaks shows linearly increases with the excitation power (see Figure S3a in the Supporting Information), which rules out the possibility of biexciton emission that would exhibit a nonlinear power response.^35^ Thus, the newly observed feature can be attributed to dark excitons. Further characterizations below similarly confirm this assignment. The appearance of the dark exciton emission band confirms a significant enhancement factor specifically of X_D_ over X_B_. As shown in Figure 1b), the out-of-plane enhancement is notably stronger than the in-plane one, demonstrating selective enhancement of the out-of-plane dark state dipole rather than the in-plane bright state. According to the previous GW theoretical calculations, the emission intensity of the dark state was predicted to be 3 orders of magnitude weaker than the bright state.^14^ However, our measurements in Figure 1d show a similar order of magnitude of the two states, indicating that a significant enhancement from the near-field configuration of the dark state is approximately 10^3^, which is consistent with the simulated results.

To further investigate the correlation between the X_D_ emission and the near-field enhancement, a retraction curve was obtained to observe the PL intensity evolution as a function of probe–sample distance.^21,36^ As shown in Figure 1e, two distinct regimes were observed. From 90 to 15 nm, the intensity of X_B_ increased linearly as the probe–sample distance decreased. This increase in signal intensity was induced by stronger far-field excitation and collection, which was also observed in retraction curves using a bare pyramid probe without the plasmonic coating (Figure S3b). In the second regime, below 15 nm, the X_D_ intensity increased sharply as the probe entered the plasmonic near-field regime, with a dimension on the order of the curvature radius of the probe, approximately 20 nm.^36^ Correspondingly, the X_B_ intensity has a relatively smaller change. This PL evolution with the probe–sample distance reveals selective near-field enhancement of the dark state emission over the bright state emission, which was also confirmed by the increase of X_D_/X_B_ intensity ratio with the decrease of ALD spacer layer as shown in Figure S3c in the Supporting Information.

It is worth noting that before the probe was in contact with the sample, the X_B_ emission started to quench, while the increase rate of the X_D_ slowed down, which can be attributed to nonradiative damping by the nanocavity.^4^ Based on this observation, the probe–sample distance at the set point (95% of free amplitude) of tuning fork vibration was estimated to be around 12 nm (see Figure S3d in the Supporting Information for more details).

In the near-field imaging experiment, the gold-coated pyramid probes scanned over the ML WSe_2_ sample at a constant probe–sample distance. The feedback signal for height sensing was set as the amplitude change, while the emission spectrum was integrated for 200 ms at each pixel with an 8 nm step size. The total time to perform the near-field scan at a dimension of 800 nm × 800 nm was approximately 55 min. To deconvolve the X_D_ and X_B_ signals, two Lorentzian peaks centered at the respective wavelength were used to fit the spectrum recorded at each pixel (see Section S4 in the Supporting Information for details). The integrated PL intensity of each peak at every pixel was calculated to generate the X_D_ and X_B_ maps.

An ML WSe_2_ sample containing nanoscale oxidation features was imaged to demonstrate the imaging capabilities of the novel near-field technique that combined a correlated shear-force image and optical mappings. The shear-force image in Figure 2a reveals a textured surface with a roughness of approximately 2 nm and a contour along the flake edge. Through nano-Auger and Kelvin probe force microscope (KPFM) measurements at the same position (see Figure S5), the textured features were identified as oxidized regions with high surface potential. These features are not present in the freshly prepared sample (Figure S1) and are a result of around 1 month oxidation under ambient conditions.

**Figure 2:**
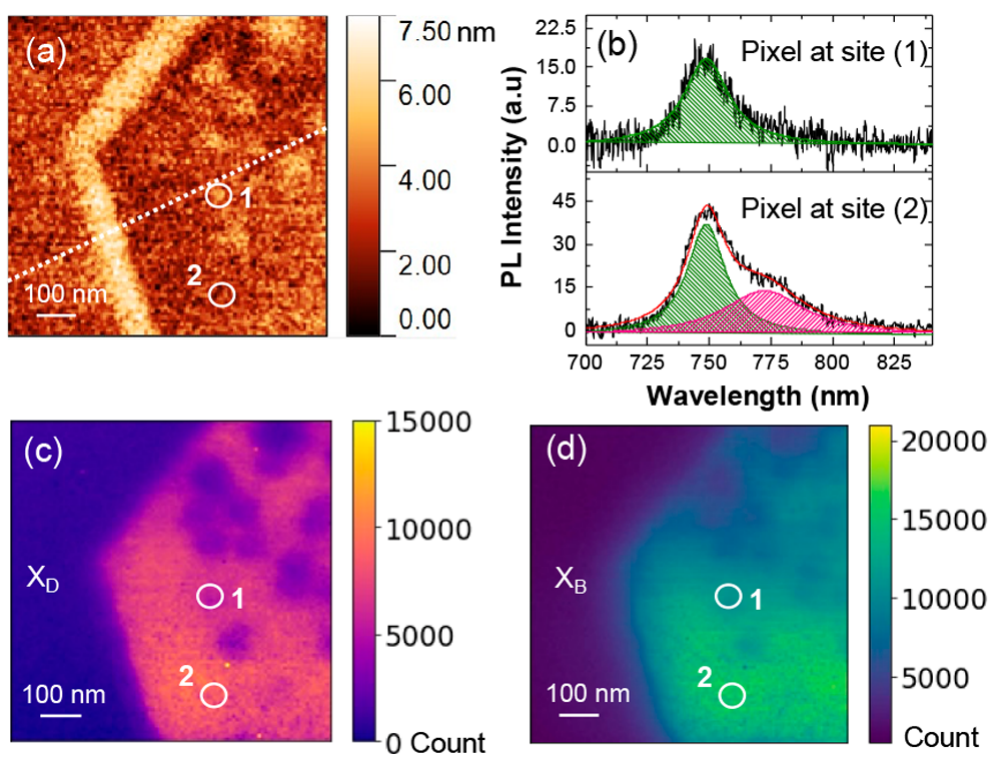
Near-field optical mapping of ML WSe_2_ containing oxidized features. (a) Shear-force image of the flake. The white dashed line is the position where the intensity profiles were plotted in the next figure. (b) Representative PL spectra of the oxidized and nonoxidized region of the flake taken at the pixel position in the sites marked as 1 and 2 in (a)–(c). Integrated dark (c) and bright (d) excitonic emission intensity mappings (800 nm × 800 nm).

The spatial resolution difference between the X_B_ and X_D_ emissions was demonstrated by comparing the recorded PL emission at oxidized and nonoxidized sites. Figure 2b shows PL spectra at these sites, with position 2 exhibiting both X_B_ and X_D_ emission, while position 1 only shows X_B_ emission. Although the oxidized sites have little to no emission, the diffraction-limited excitation and collection around the probe result in nonprobe-enhanced X_B_ emission being collected from outside of the near-field spot. For this reason, X_B_ maps cannot resolve smaller oxidized regions, unlike the X_D_ maps. Figure 2c,d display intensity maps of X_D_ and X_B_, respectively. The nonemissive oxidized features and the flake edge can be clearly identified in the X_D_ map at the corresponding position in the height image, whereas these features are less clear in the X_B_ map. Figure 3 displays the intensity profile tracked along the flake edge (marked by the dashed line in Figure 2a). The optical resolutions of the X_D_ and X_B_ maps were determined by using the standard 90–10 method,^37^ which were ∼50 and ∼200 nm, respectively. This confirms that this gap-mode-based configuration provides a spatial resolution approximately equal to the apex radius of the probe on the out-of-plane X_D_ map which was ∼4 times higher than that of the X_B_ map (see Figure S6 in the Supporting Information for the addition results confirming this high-resolution contrast).

**Figure 3:**
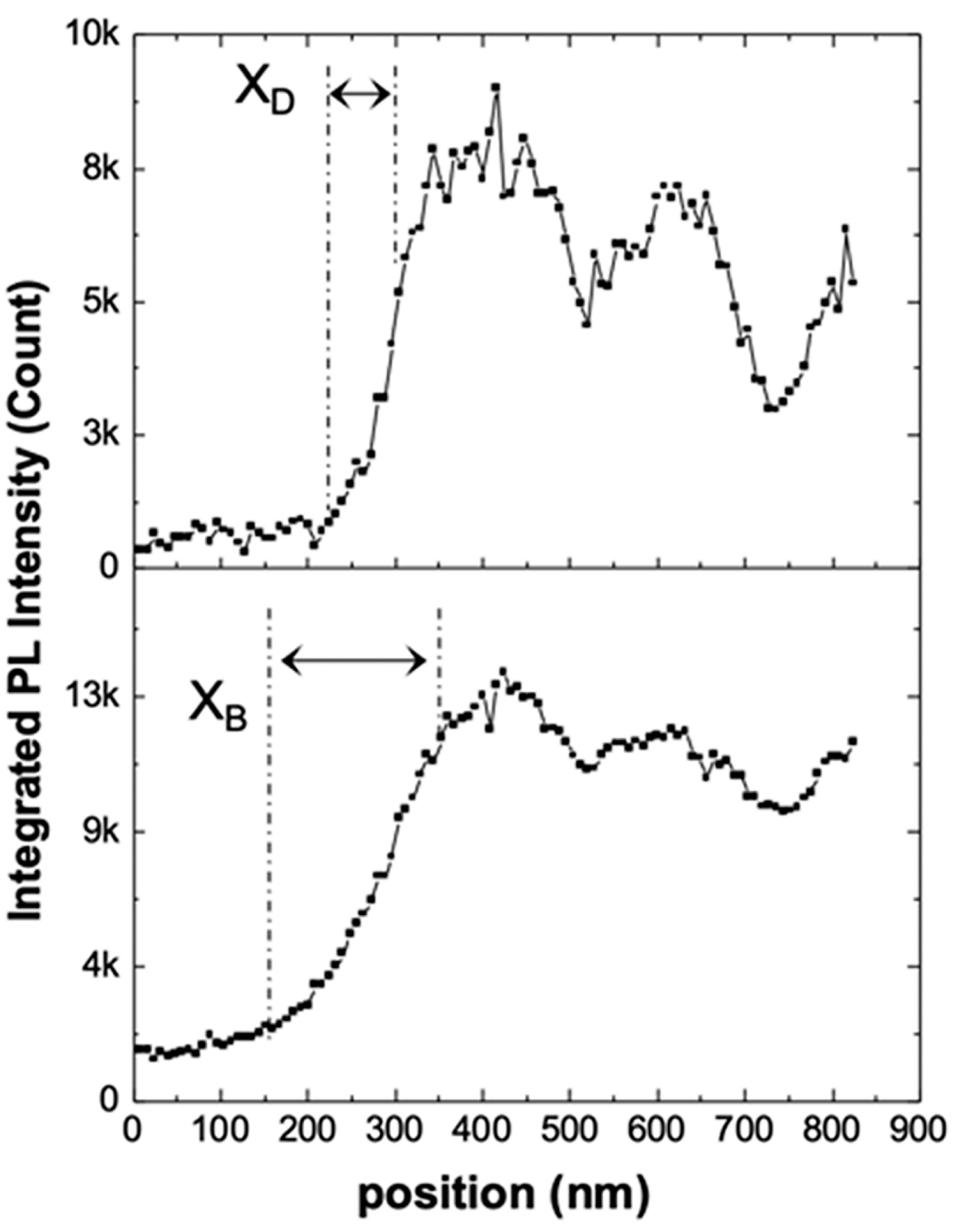
Comparison of the spatial resolution between X_D_ and X_B_ along the white dashed line marked in Figure 2.

The near-field technique was also applied to map regions of nanobubbles in the ML WSe_2_ samples. These nanobubbles are generated during sample preparation under ambient conditions where water molecules, air, or nanoparticles can be trapped between the ML and substrate.^38,39^ Such bubbles have emerged as an important means of engineering the excitonic states, through potential modulations of localized strain^11^ or alterations in doping concentration.^39^

The height maps in Figure 4a,b show four distinct areas: The flat area (area 1) where WSe_2_ was in contact with the substrate, two bubble sites (areas 2 and 4), and the adjacent region (area 3) which appeared to be suspended, as indicated by its disappearance after the near-field experiments, which was confirmed in the AFM image in Figure S7.2a. Notably, the height change in area 3 was not captured in the shear-force image (Figure S7.1a) in the rapid raster scan conducted prior to the experiments, likely due to the reduced height sensitivity of the high-speed scan. Consequently, the suspended region with reduced support cannot be captured accurately in the shear-force image.

**Figure 4:**
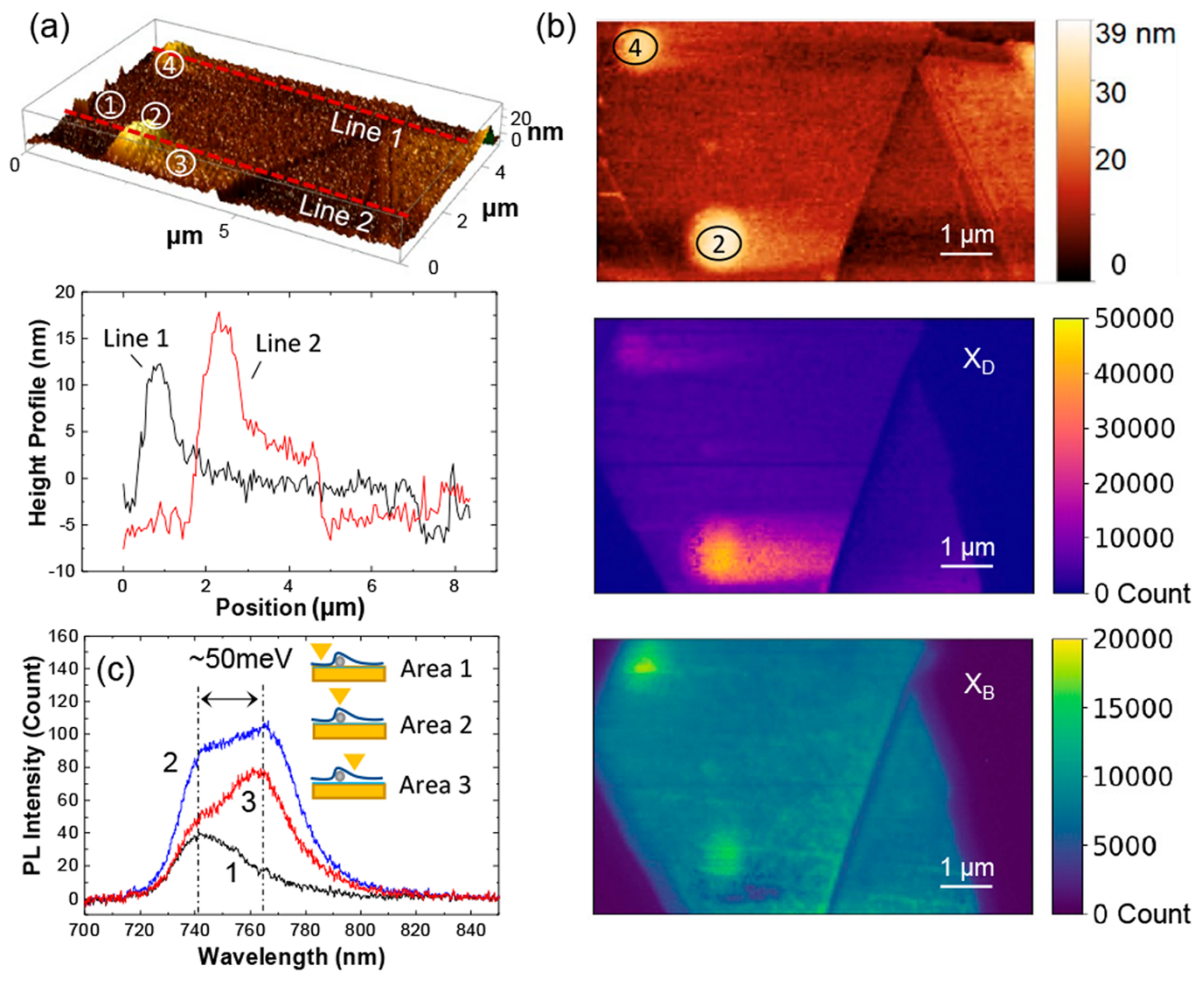
Dark exciton enhancement on the nanobubble areas. (a) 3-dimensional shear-force image recorded by the fiber probe scan and the corresponding height profiles along the red dotted lines. The four distinct areas 1, 2, 3, and 4 are the flat region, a nanobubble, the suspended region, and the second nanobubble. (b) 2-dimensional shear-force image and correlated dark exciton and bright exciton map with an ∼50 nm step size. Scale bars: 1 μm. (c) Representative PL spectra extracted from the flat (Area 1), strained (Area 2), and suspended regions (Area 3).

As shown in Figure 4b, it was observed that the bubble sites and area 3 exhibited stronger X_B_ emission compared to the flat area. This enhanced emission was also observed in far-field imaging using an objective lens, as well as the results by the as-imprinted pyramid probe without the gold coating (see Figure S8). However, when the coated probe was used, it was found that enhanced X_D_ emission was also present in these sites, which is not accessible through the far-field approach. This enhanced X_D_ was further confirmed by repeated near-field mapping in area 4 using a different probe (see Figure S7.2 in the Supporting Information).

Figure 4c displays representative spectra taken from the flat area (1), bubble (2), and suspended area (3), showing minimal energy shifts for both the X_B_ and X_D_ exciton peaks. This suggests that the strain effect in these areas is relatively small, consistent with the estimated strain of less than 0.1% associated with the geometric structure of the bubble in area 4 (see Section S9 in the Supporting Information for calculation details). The enhanced X_B_ and X_D_ emission observed at the bubble sites can be attributed to the lifting of contact with the substrate, which reduces the potential substrate dopant effect,^40^ and the other dielectric screening effects.^41^ This is further supported by the reduced emission observed in area 3 in the optical maps obtained during the high-speed scan (Figure S7.1a) and the scan using the bare pyramid probe scan (Figure S8b), where no significant height changes in area 3 were observed.

Additionally, a scan on smaller bubbles is shown in Figure S10, revealing an energy shift in the bright states and a larger energy difference between the X_B_ and X_D_ emissions (see Section S10 in the Supporting Information). This observation aligns with recent studies highlighting the sensitivity of X_D_ emission to strain. For instance, Kim et al.^9^ have reported a correlation between strain-induced excitonic emission and X_D_, with lifetimes reaching tens of nanoseconds. Gelly et al.^19^ have shown that dynamic control of strain can facilitate the funneling of X_D_ and increase the emission. Moreover, Hasz et al.^10^ reported an increase in the X_D_ emission with increasing strain using a scattering-type near-field probe at the nanobubble sites. These findings further underscore the significance of our technique in investigating dark excitons.

In conclusion, we have successfully used a novel near-field configuration to perform hyperspectral near-field mapping of spin-dark excitonic emission in ML WSe_2_. The near-field configuration, which consists of a pyramidal probe coated with a thin gold film and gold substrate, offers distinct advantages over the traditional scattering-type probes. Such a reduced interaction with the excitation laser promotes a stable TEPL scan and provides an excitation profile that minimizes the far-field background. Both experimental and theoretical results confirm the capacity of the near-field configuration to selectively enhance X_D_ emission within an optical volume consistent with the gap spacing. The high contrast in the spatial resolutions between the dark and bright states provides independent optical access to X_D_ and consequently a direct analysis of the local oxidized features and bubble sites. Our study provides experimental proof that the formation of nanobubbles can enhance the X_D_ emission efficiently, which was previously inaccessible with far-field optical mapping. These findings align with recent research efforts^9,19^ on the impact of localized strain on dark state emission, which aims to better understand the photophysics of the strain localized quantum emitter. Overall, our approach offers an alternative method for TEPL on 2D materials and has broad implications for probing the local excitonic emission in these materials.

## Data Availability

The data that support the findings of this study are available from the corresponding authors upon reasonable request.

## Supporting Information Available

Sample preparation, simulation details, further details on the Purcell effect on the dark excitonic emission, spectral fitting, nano-Auger data, additional hyperspectral images, far-field optical mappings on the nanobubble areas, characterization data before and after the near-field measurement on the nanobubble areas, strain calculation details, additonal data on the nanobubbles (PDF)
