# Supplementary material for: Near-Field Coupling with a Nanoimprinted Probe for Dark Exciton Nanoimaging in Monolayer WSe2
Source: Nano Lett. 2023 Jun 1;23(11):4901–7. doi: 10.1021/acs.nanolett.3c00621 (PMC10273309; doi:10.1021/acs.nanolett.3c00621)
Supplement: Supplementary file 1 — nl3c00621_si_001.pdf [file nl3c00621_si_001.pdf]

Supporting Information for

Near-field Coupling with a Nanoimprinted Probe for Dark Exciton Nanoimaging in Monolayer WSe_2

Junze Zhou[1], John C. Thomas[1], Elyse Barre[1], Edward S. Barnard[1], Archana Raja[1], Stefano Cabrini[1], Keiko Munechika[2], Adam Schwartzberg[1*], Alexander Weber-Bargioni[1*]*

[1]The Molecular Foundry, Lawrence Berkeley National Laboratory, 1 Cyclotron Road, Berkeley, CA 94720, USA

[2]HighRI Optics, Inc. 5401 Broadway Ter 304, Oakland, CA 94618, USA

** Corresponding Authors: junzezhou@lbl.gov, amschwartzberg@lbl.gov, afweber-bargioni@lbl.gov*

Section 1: Sample preparation

A 120 nm gold thin film was first deposited directly on a prime-grade silicon wafer with the e-beam evaporator (Semicore SC600). A square glass chip was then glued to the free surface of the gold thin film using Ormocomp photoresist. After UV exposure for 90 seconds, the gold was firmly attached to the UV-cured Ormocomp photoresist, enabling the gold to be peeled off from the silicon wafer. The top surface of the gold on the glass chip has a roughness below 0.5 nm, as shown in the height profile in the substrate region in Figure S1 (a). A ~2 nm SiO_2 spacer layer was deposited on the top surface using Atomic Layer Deposition (ALD). To prepare the monolayer (ML) WSe_2, the WSe_2 crystal was thinned down by using tape a few times until the color of the thin crystal looks reddish. The WSe_2 was then mechanically exfoliated on the SiO_2-coated gold substrate. The monolayer region was identified in the optical microscope by checking the color contrast between the substrate and the flake. The thickness of the layer was further confirmed by a single layer thickness of around 0.8 nm in the AFM height image (Figure S1 (c)), and the strong PL emission peak at around 750 nm[1] (Figure S1 (b) and (d)).

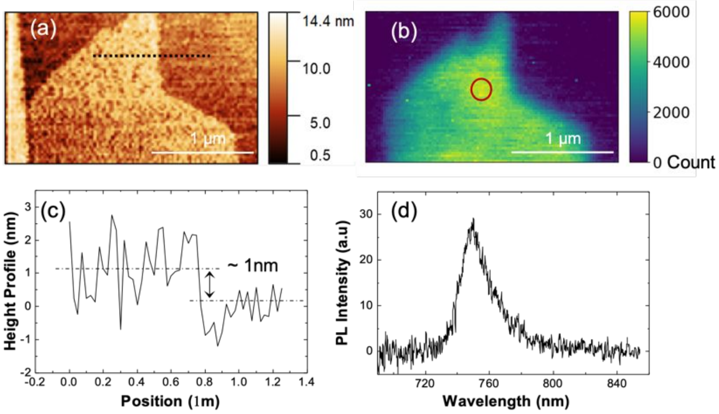

Figure S1. Height and emission images of the monolayer WSe_2 on SiO_2-coated gold substrate. (a) and (c) are the height map and the height profile along the dotted line. (b) and (d) is the PL map and the PL spectrum at the circular marked position in (b).

Section S2: Numerical Simulation

The simulation of the detection and excitation profiles in Figure 1 (b) and (c) were conducted using the commercial Finite-Difference Time-Domain (FDTD) method based software, Ansys Lumerical FDTD. The relative permittivity of gold $\varepsilon(\omega) = \varepsilon_r - i\varepsilon_r$ used for simulation was taken from Johnson and Christy's report, and the refractive index of the Ormocomp and SiO_2 were set as $n = 1.5$. The simulations were performed in 2 dimensions since the light propagations in both cases are symmetric in the in-plane direction (perpendicular to the plane of incidence). To simulate the probe, a cone structure with a curvature size of 20 nm, a taper angle of ~70°, an Au coating of 20 nm, and a height of 5.6 µm, was used. The probe is placed 15 nm above the gold substrate. The objects were placed in the center of a volume with a refractive index $n = 1$, surrounded by 16 layers of stretched coordinate Perfect Matched Layers (PML) on 4 sides to avoid unphysical reflections from the sides. All PMLs were placed far away from the objects to avoid spurious effects from a potential interaction between the evanescent waves and the PML. A mesh with a 1 nm step size in both horizontal and vertical directions was used at the apex of the probe to guarantee accuracy and mesh-independent results.

For the detection profile simulations, dipole emitters with an out-of-plane and in-plane polarization are placed 2 nm away from the gold substrate. The monitor was placed inside the pyramid to record the transmitted light intensity during the sweeping of the dipole position. The distance range was -250 nm to 250 nm for the out-of-plane oriented dipole and -900 nm - 900 nm for the in-plane oriented dipole.

In the simulation of the light profiles at the probe, a plane wave with an electric field component $E_y = 1V/m$, in the direction parallel to the axis of the fiber. This was chosen due to the dominant nature of the linearly polarized transverse mode in the single-mode fiber. The light intensity profile was recorded by a monitor placed 12 nm away from the probe.

Section S3: Purcell enhancement of Dark exciton

This section provides additional information on our near-field configuration and the visualization of the dark state via the Purcell effect. Figure S3 (a) shows that the power dependence of the deconvoluted X_B and X_D emission can be fitted with linear curves, excluding the possible contribution of the bi-excitonic emission. Figure S3 (b) shows probe-sample distance dependence of emission peak intensity using the bare (uncoated) pyramid tip. A smooth increase was observed when the probe-sample distance was less than around 270 nm before landing, and no sharp increase and quench were observed. In Figure S3 (c), we confirm the gap-size dependent enhancement of the X_D emission by showing its dependence on the thickness of the spacer layer. We found that only when the deposition cycle of Al_2O_3 is less than 30 cycles (~3 nm), the X_D is visible in the spectra, and the X_D/X_B intensity ratio increase as the thickness decreases. Together with the retraction curve in the main text, these gap distance dependent X_D emission results provide further support that the Purcell enhancement of X_D in our near-field configuration. In Figure S3 (d), we explain how we define the probe-sample distance, which we estimated to be around 12 nm. This estimate was obtained by comparing the position of the probe at the set point (around 95% of the free magnitude) to the position when the PL emission of the bright exciton starts to quench. This change in position signifies that the probe had entered the quantum tunneling regime, which has been reported to have a distance range of less than 2 nm.[2] Therefore, our estimated value of 12 nm for probe-sample distance was obtained from this comparison. While the precise determination of probe-sample distance using shear-force microscopy can be challenging, this estimate is a relatively accurate representation of our experimental conditions.

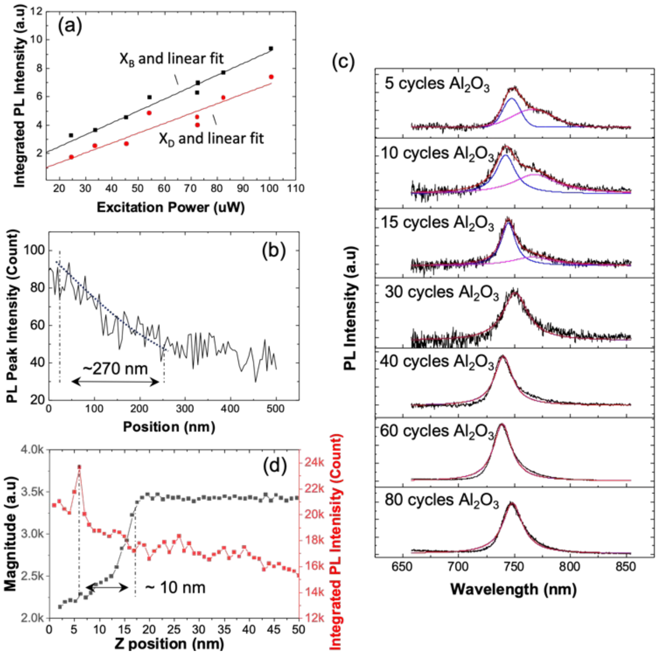

Figure S3. (a) Power dependence of the TEPL intensity of the X_B and X_D emission. The lower line is a fit of the X_D emission exhibiting a linear power dependence. (b) Tip-sample distance dependence of emission peak intensity using the bare (uncoated) pyramid tip. (c). PL spectra evolution as a function of the Al_2O_3 spacer layer thickness, demonstrating polarization-sensitive Purcell enhancement of the X_D emission. (d). Evolution of the bright exciton emission signal and the corresponding magnitude change of the tuning fork. ~10 nm is the distance change observed as the probe moves from the set point (95% of free amplitude) to the position where the X_B emission starts to quench.

Section S4: Hyperspectral Image Reconstruction

The spectral fitting was conducted pixel-by-pixel within an acquired dataset using the Python lmfit package[3]. The fitting employed two Lorentzian peaks, with the X_B peak fixed at 750 nm and a width of 20 nm, as determined from the normalized spectrum obtained by the uncoated pyramid tip (Figure 1d). The intensity of both X_D and X_B were left to be fitted, with the only further numerical constraints being the wavelength and width range of X_D, which were set to 770 - 778 nm and 20 - 50 nm, respectively. This fitting method allowed for consistent modeling of all hyperspectral datasets, which typically consisted of several thousand individual spectra. The deconvolved peaks were then summed over a given intensity range and input into a dimensionally matching matrix for visualization and further analysis.

Section S5: Nano-Auger and KPFM

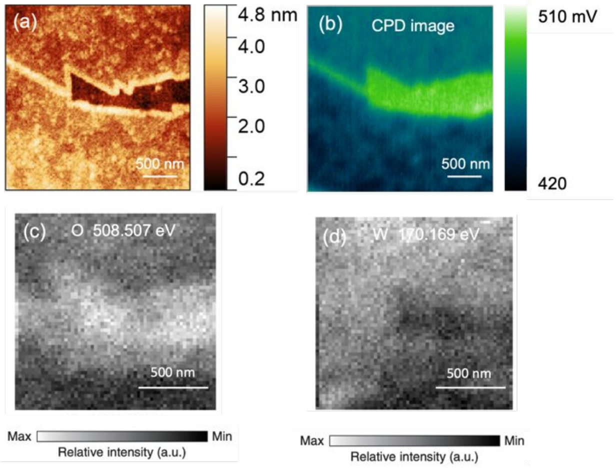

Figure S5. Identification of oxidation contour along the flake. (a), (b) AFM height and correlated contact potential difference (CPD) images. The CPD image shows the contour with a width of ~50 nm along the edge of the flake, has higher surface potential than the ML WSe_2. (c), (d) Elemental mapping of Oxygen (c) and Tungsten (d) based on O KLL (508.507 eV) and W MNN (170.169 eV) Auger transition. The contour of the flake is O-rich while the W composition is almost uniform over the flake.

Section S6: Additional scans

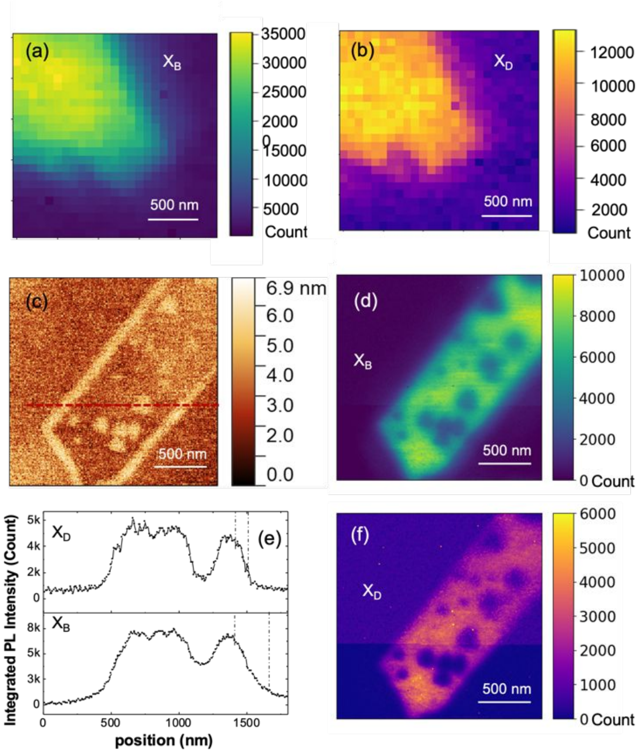

Figure S6. Deconvoluted X_B and X_D maps on the different samples using different probes. Deconvoluted X_B (a) and X_D (b) map along the same flake edge. (c), (d), and (f) are shear-force image and deconvoluted X_B and X_D maps along the same oxidized flake. (e) The optical resolution of X_B and X_D map along the dotted line marked in (c).

Section S7: Characterization before and after the near-field measurement

Figure S7.1 displays the data obtained from a rapid scan conducted prior to Figure 4 in the manuscript, which revealed similar emission intensity values in both nanobubbles. It is worth noting that the intensity difference presented in Figure 4 may have been caused by system drift during the long raster scan, which started from the bottom left. Figure S7.2 shows the repeated near-field measurements in Area 4 using a different gold-coated probe, where the dark states enhanced by the strain are reproducible.

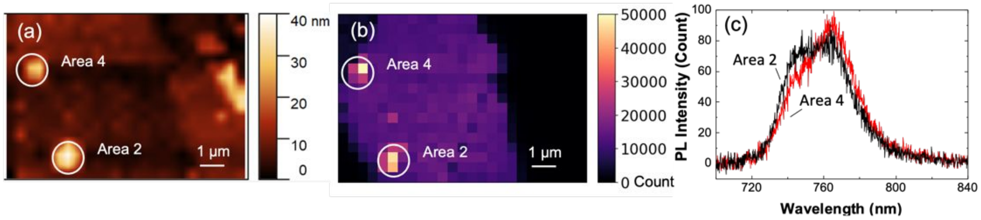

Figure S7.1. Characterization of the nanobubbles using a rapid raster scan to localize the nanobubble areas. (a) Shar force image and correlated PL emission map (b) using a rapid raster scan (pixel step of 400 nm) prior to the near-field experiment. The suspended area 3 was not identified, likely due to the reduced height sensitivity in the high-speed scan. (c) Spectra from areas 2 and 4.

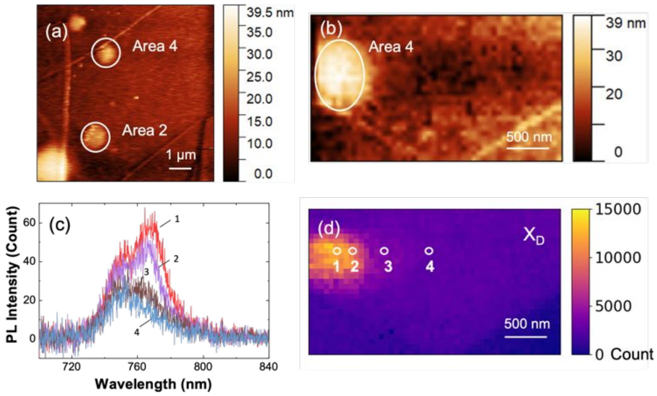

Figure S7.2. Reproducibility of the near-field measurement at the nanobubble in Area 4. (a). AFM image using the cantilever-type commercial AFM probe (ATEC-NC) after the near-field measurement. The height change in Region 3 (see Figure 4 a) cannot be identified. (b) Shear-force image contains the bubble of Area 4. (c) TEPL spectra in the corresponding position 1, 2, 3, 4 as marked in (d). (d) The X_D map shows that the enhanced X_D at the bubble site is reproducible.

Section S8: Far-field characterizations of the nanobubbles areas.

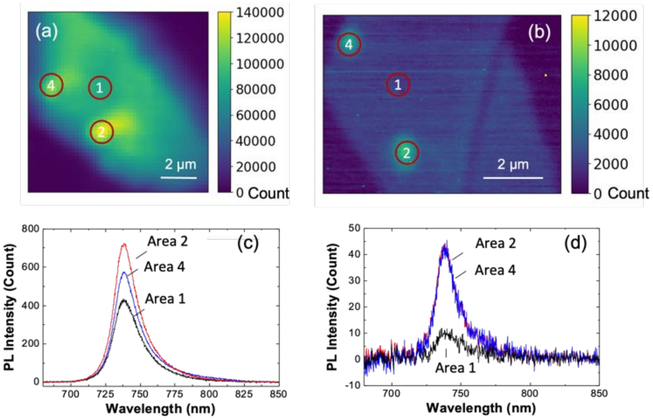

Figure S8. Emission maps detected through the objective lens (x100, NA 0.7) (a) and bare pyramid tip (b), the flat region and nanobubble areas are marked as 1, 2, and 4 to align with the label in the manuscript. (c) and (d) are PL spectra of the marked position in (a) and (b), where only the emissions of the X_B were evidenced.

Section S9: Estimation of strain

The strain that induced enhanced dark excitonic emission ~0.08%, as estimated using the following equation:[4] $\varepsilon = \frac{\pi^2 \delta h}{\lambda^2(1-\sigma^2)}$, where δ is the area, height (of ~20 nm), h is the layer thickness (of ~1 nm), λ is the bubble width (of ~ 500 nm), and σ is 0.19, the Poisson's ratio of WSe_2,[5] respectively.

Section S10: Dark exciton mapping in nanobubble (~0.16% strain)

We utilized the near-field technique to investigate a new sample configuration, consisting of a WSe_2 monolayer separated from a gold substrate by a spacer layer of ~5 nm thick hBN flake. With this sample, we imaged a smaller nanobubble compared to those in Figure 4. By analyzing the shear-force image in Figure S10 (a), we estimated the strain present in bubble 1 to be ~0.16%, using measured parameters such as the height of the bubble (~10 nm) and its width (~250 nm). Comparing the spectra obtained from bubble 1 and the adjacent flat region 2, we observed a shift in the emission of the bright states by approximately 15 meV. This shift aligns well with the estimated strain, as it has been reported that the bandgap of the 2D materials decreases at approximately 100 meV/% for uniform biaxial strain.[6] Furthermore, we observed a relatively large dark-bright band splitting of approximately 72 meV within bubble 1. This splitting can potentially be attributed to changes in the momentum dark excitonic emission, as suggested by a recent theoretical paper.[7] Alternatively, it may be influenced by the presence of charged dark states.[8] These possibilities warrant further investigation and provide avenues for future research.

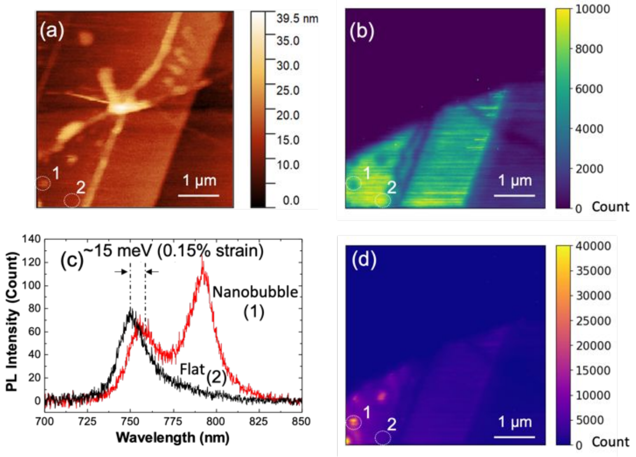

Figure S10. Dark exciton imaging at nanobubble with a strain of ~0.16%, estimated from the measured height change in the shear-force image. (a) Shear-force image of the sample, which cycle 1 representing the bubble and cycle 2 representing the nearby flat region. (b) Emission map integrated within the wavelength range of 670 nm- 760 nm. (c) Representative spectra from the nanobubble site (1) and nearby flat region (2), showing a redshift of the bright states by ~15 meV, corresponding to a strain of ~0.15% based on the predicted decrease in the bandgap. (d) Emission map integrated within the wavelength range of 760 nm- 840 nm.

Reference

(1) Wang, G.; Chernikov, A.; Glazov, M. M.; Heinz, T. F.; Marie, X.; Amand, T.; Urbaszek, B. Colloquium: Excitons in Atomically Thin Transition Metal Dichalcogenides. *Rev. Mod. Phys.* 2018, *90* (2), 021001.

(2) Kravtsov, V.; Berweger, S.; Atkin, J. M.; Raschke, M. B. Control of Plasmon Emission and Dynamics at the Transition from Classical to Quantum Coupling. *Nano Lett.* 2014, *14* (9), 5270–5275.

(3) Newville, M.; Stensitzki, T.; Allen, D. B.; Rawlik, M.; Ingargiola, A.; Nelson, A. Lmfit: Non-Linear Least-Square Minimization and Curve-Fitting for Python. *Astrophysics Source Code Library*. June 1, 2016, p ascl:1606.014.

(4) Koo, Y.; Kim, Y.; Choi, S. H.; Lee, H.; Choi, J.; Lee, D. Y.; Kang, M.; Lee, H. S.; Kim, K. K.; Lee, G.; Park, K.-D. Tip-Induced Nano-Engineering of Strain, Bandgap, and Exciton Funneling in 2D Semiconductors. *Adv. Mater.* 2021, *33* (17), e2008234.

(5) Kang, J.; Tongay, S.; Zhou, J.; Li, J.; Wu, J. Band Offsets and Heterostructures of Two-Dimensional Semiconductors. *Appl. Phys. Lett.* 2013, *102* (1), 012111.

(6) Lloyd, D.; Liu, X.; Christopher, J. W.; Cantley, L.; Wadehra, A.; Kim, B. L.; Goldberg, B. B.; Swan, A. K.; Bunch, J. S. Band Gap Engineering with Ultralarge Biaxial Strains in Suspended Monolayer MoS2. *Nano Lett.* 2016, *16* (9), 5836–5841.

(7) Feierabend, M.; Khatibi, Z.; Berghäuser, G.; Malic, E. Dark Exciton Based Strain Sensing in Tungsten-Based Transition Metal Dichalcogenides. *Phys. Rev. B.* 2019, *99* (19), 195454.

(8) Rahaman, M.; Selyshchev, O.; Pan, Y.; Schwartz, R.; Milekhin, I.; Sharma, A.; Salvan, G.; Gemming, S.; Korn, T.; Zahn, D. R. T. Observation of Room-Temperature Dark Exciton Emission in Nanopatch-Decorated Monolayer WSe_2 on Metal Substrate. *Advanced Optical Materials*. 2021, 9 (24), 2101801.
